# Supermicrosurgery: History, Applications, Training and the Future

**DOI:** 10.3389/fsurg.2018.00023

**Published:** 2018-03-21

**Authors:** Ido Badash, Daniel J. Gould, Ketan M. Patel

**Affiliations:** ^1^Keck School of Medicine of USC, University of Southern California, Los Angeles, CA, United States; ^2^Division of Plastic and Reconstructive Surgery, University of Southern California, Los Angeles, CA, United States

**Keywords:** supermicrosurgery, microsurgery, plastic surgery, free flap, perforator, soft tissue reconstruction, lymphedema, lymph node transfer

## Abstract

Supermicrosurgery, a technique of dissection and anastomosis of small vessels ranging from 0.3 to 0.8 mm, has revolutionized the fields of lymphedema treatment and soft tissue reconstruction. The technique offers several distinct benefits to microsurgeons, including the ability to manipulate small vessels that were previously inaccessible, and to minimize donor-site morbidity by dissecting short pedicles in a suprafascial plane. Thus, supermicrosurgery has become increasingly popular in recent years, and its applications have greatly expanded since it was first introduced 20 years ago. While supermicrosurgery was originally developed for procedures involving salvage of the digit tip, the technique is now routinely used in a wide variety of microsurgical cases, including lymphovenous anastomoses, vascularized lymph node transfers and perforator-to-perforator anastomoses. With continued experimentation, standardization of supermicrosurgical training, and high quality studies focusing on the outcomes of these novel procedures, supermicrosurgery can become a routine and valuable component of every microsurgeon’s practice.

## Introduction

Recent advances in technology and refinement of surgical techniques have ushered forth a new age in the field of reconstructive microsurgery. Microsurgical procedures have evolved from simple soft tissue reconstruction to more sophisticated applications of these techniques, including vascularized lymph node transfer (VLNT) and perforator-to-perforator anastomosis (1, 2). The outcomes and complication rates from microsurgery have improved over the past few decades, allowing today’s microsurgeons to focus more on minimizing donor-site morbidity and maximizing the function and aesthetics of the reconstruction (3). Most importantly, today’s reconstructive surgeons are able to execute procedures that were previously unachievable, such as anastomosis of vessels with a caliber less than a single millimeter. These advanced procedures have been grouped under the field of supermicrosurgery (4, 5).

Supermicrosurgery is defined as a technique of microneurovascular anastomosis for vessels and single nerve fascicles of 0.3 to 0.8 mm (4). The surgical technique utilizes highly delicate microsurgical instruments (30- to 80-micron needle micro-sutures) to accomplish small anastomoses (6). First introduced 20 years ago, the technique was pioneered by Koshima et al. in reconstructions involving vessel repair in the distal finger. Since then, the technique has been used in reconstructions that were previously limited by vessels of small caliber. For example, supermicrosurgery has allowed for the use of perforator-to-perforator freestyle flaps in diabetic foot wounds with compromised vasculature and lymphovenous anastomosis (LVA) for obstructive lymphedema (7, 8). Supermicrosurgical techniques have expanded the possibilities of what was previously achievable within the field of microsurgery.

This review summarizes the history of the development of supermicrosurgery, current applications of this technique, training methods and future directions for supermicrosurgery. We have searched the PubMed database for articles published in the English language. Keywords used included “supermicrosurgery,” “perforator flaps,” “perforator-to-perforator,” “reconstructive microsurgery,” “lymphedema,” “microvascular anastomosis,” and “microsurgery simulation.” Original and review articles related to the applications of supermicrosurgery were reviewed. A total of 106 articles were reviewed and included, with levels of evidence ranging from I to V (Table 1) (9).

**Table 1:** Levels of Evidence for Therapeutic Studies.

Level of Evidence	
I	Systematic review (with homogeneity) of randomized control trials (RCT); individual RCT (with narrow confidence intervals); all or none study.
II	Systematic review (with homogeneity) of cohort studies; individual cohort study (including low quality RCT, e.g. <80% follow-up); “outcomes” research; ecological study.
III	Systematic review (with homogeneity) of case-control studies; individual case-control study.
IV	Case series (and poor quality cohort and case-control study).
V	Expert opinion without explicit critical appraisal or based on physiology, bench research or “first principles”.

## History of Supermicrosurgery

The field of microsurgery dates back to the 1960s, when new surgical techniques and intraoperative magnification were introduced into vascular surgery through a series of landmark experimental procedures (10). The first microvascular surgery is reported to have occurred in 1960, when JH Jacobson and EL Suarez successfully joined two ends of a canine carotid artery together under a microscope (11). A number of pioneers around the world subsequently began developing the techniques and tools that would evolve into microvascular surgery. Eventually, this technology would be used to successfully to replant amputated digits and hands in patients for the first time (12). It was during this period that Harry J. Buncke performed his now famous experiments involving tissue transplantation in animal models, and developed many of the central principles of microsurgery that have led to his designation as the “founding father of microsurgery” (10).

Buncke and the other microsurgical pioneers of the 1960s, including John Cobbett, James Smith and Susumu Tamai, held the first ever panel on microsurgery at the Annual Meeting of the American Society of Plastic and Reconstructive Surgeons in New York City in 1967 (10). This meeting marked the first in-person exchange of microsurgical techniques, which included peripheral nerve repairs, digit transfers and tissue transplantation. Following the success of this meeting, the International Microsurgical Society was founded in 1970, and held the first international workshop for microvascular transplantation in the same year (10). Thus, over, the course of a decade, the field of microsurgery was born.

In 1987, a new era of microvascular reconstruction emerged when Taylor and Palmer provided a whole body perforator map that enabled surgeons to perform safe skin flaps using perforating vessels (13). Koshima et al. then published the first use of a perforator flap in 1989, the deep inferior epigastric skin flap, which heralded the development of a variety of different perforator flaps in the years after (14). The possibility of utilizing skin flaps based on “perforasomes” distributed throughout the body created a new paradigm in free flap reconstruction, allowing surgeons greater freedom when choosing which flaps to use (15).

Building on the concept of perforator flaps, Koshima reported the first use of flaps based on perforating vessels with a caliber less than 0.8 mm at the First International Course on Perforator Flap and Arterialized Skin Flaps in 1997. The possibility of utilizing such small vessels for anastomosis was significant, as this greatly expanded surgeon freedom in choosing free tissue flaps while also decreasing donor site morbidity through preservation of fascia, muscles, nerves and major vessels in the process of dissection. Koshima et al. called this technique “supramicrosurgery” for the first time in their description of the paraumbilical perforator flap in 1998 (16). In 2007, Koshima referred to the technique as “supermicrosurgery” at the first international meeting on microsurgical innovative technology, and published a definition of the procedure in 2010 (5). A consensus wasreached on the name of “supermicrosurgery” at the First European Conference on Supermicrosurgery held in Barcelona in March 2010 (4). The technique was quickly adopted by surgeons around the world, and several studies began reporting successful use of perforator flaps and other microvascular procedures utilizing vessels of small caliber (<0.8 mm) (17–21). The use of supermicrosurgery thus developed as the next step in the evolution of microvascular reconstruction.

Over the next decade, supermicrosurgical techniques became increasingly incorporated into the surgical toolkit of microsurgeons. Supermicrosurgery has revolutionized the management of lymphedema, and the technique has also helped salvage fingertip replantations and free flap reconstructions (22–24). Furthermore, the advent of supermicrosurgery and subsequent flap customization with freestyle flaps has allowed reconstruction to not only address soft-tissue coverage, but also functional recovery, chronic infected wound control, and cosmetic improvement (19). The utility of supermicrosurgery allows for another level of operating freedom from which to craft reconstructions. The following sections outline some of the current applications and controversies in its use.

## Current Applications

### Lymphedema Treatment

Supermicrosurgery has revolutionized the field of lymphedema, a chronic condition of the lymphatic system resulting in the collection of protein-rich fluid in the interstitial space that significantly impairs patient quality of life (25–30). Until recently, lymphedema was predominantly managed with nonsurgical physiotherapy involving compression wrapping, manual drainage and exercise, although liposuction and debulking surgery have also been used with varying degrees of success (29). The advent of supermicrosurgery introduced the procedures of LVA and supermicrosurgical VLNT, which are becoming increasingly utilized treatment options for this condition (1, 8, 31).

**Figure 1:**
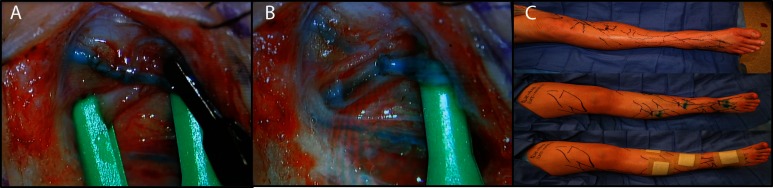
Lymphovenous anastomosis by end-to-side technique. The lymphatic vessel and vein are shown clamped **(A)**. After anastomosis, the outflow vein and lymphatic vessel are unclamped to demonstrate flow **(B)**. Many lymphovenous anastomoses are performed on each limb in sequence in order to improve lymphatic outflow **(C)**.

LVA involves the creation of a microsurgical connection between subcutaneous lymphatic vessels and venules in order to bypass lymphatic drainage in the distal extremity. The anastomosis may be performed through either an end-to-end, end-to-side, or side-to-end technique (32) (Figure 1). Lymphatic vessels in the distal extremity are used since distal lymphatics are less affected by lymphedema and are thus more readily available for bypass. Additionally, subcutaneous lymphatics and venules are the preferred vessels for LVA, as venous pressure is lower in subcutaneous venules and results in less venous backflow (3). Successful LVA requires supermicrosurgical techniques, as lymphatic vessels have a small vessel caliber often measuring less than 0.5 mm. Thus, while LVA was originally reported in the 1960s, it has only been performed consistently and successfully since the advent of supermicrosurgery (25).

Koshima et al. first reported their experience performing LVA using supermicrosurgery in 1996 (33). Since then, the technique has been utilized for lymphedema treatment in various institutions worldwide and has gained significant traction as a major option for surgical management of lymphedema (34). Recent innovations in imaging techniques have allowed surgeons to better evaluate lymphatic vessel patency and function, permitting more reliable planning and improved long-term patency of the anastomosis (25). For example, a study by Campisi et al. investigating 1,800 patients with peripheral lymphedema treated with LVA found subjective improvement in 87% of patients and objective volume changes in 83%. Importantly, of the patients followed up in their study, 85% were able to discontinue the use of conservative measures, with an average follow-up time of 10 years and a 69% average reduction in excess volume (35). Other studies have similarly reported positive quantitative and qualitative improvements in lymphedema following LVA (31, 32, 36–39). The introduction of additional techniques and refinement of the procedure will likely continue to improve outcomes in the future (39).

Nonetheless, the use of LVA for lymphedema treatment remains controversial. Although some study results have been encouraging, other studies have reported less favorable outcomes, including a lack of volume reduction or benefits over nonoperative management (40). LVA is a challenging technique that requires a high level of technical skill to perform, the benefits are not always visible, and an objective method for the evaluation of results is still lacking (34). These drawbacks, combined with the small number of high quality studies and lack of clinical algorithms for lymphedema treatment, have led to skepticism regarding the efficacy and role of the LVA technique. For now, it is thought that the best indication for LVA is early stage lymphedema resistant to nonoperative treatment (25, 41).

**Figure 2:**
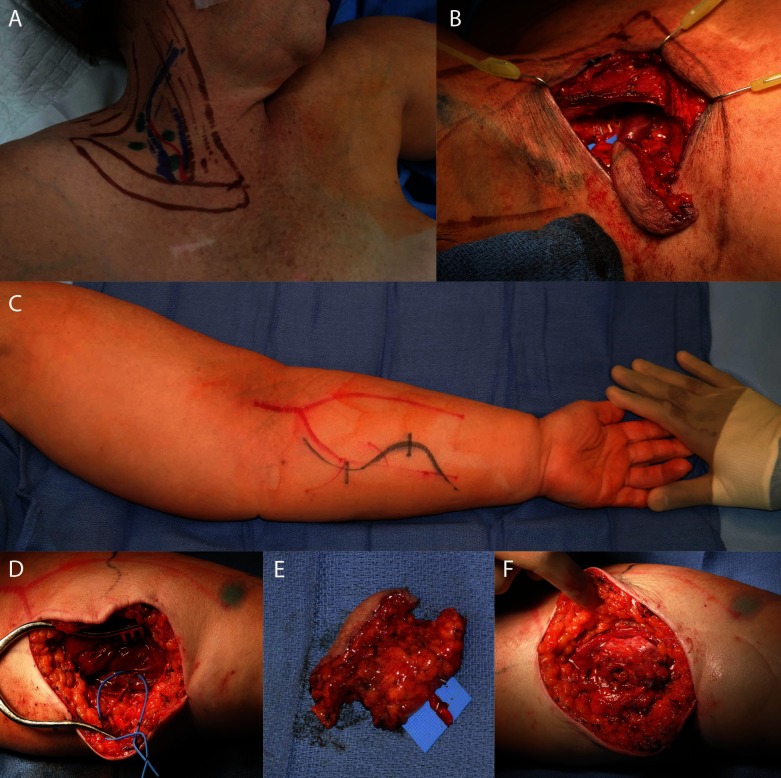
Supraclavicular flap. Skin markings for the supraclavicular donor site of the vascularized lymph node transfer to the upper extremity **(A)**. Nodes were confirmed with lymphoscintigraphy and ICG. **(B)** shows the flap in place after harvest. **(C)** shows the recipient edematous upper extremity. **(D)** shows the dissected recipient artery and vein. **(E)** shows the dissected flap, and **(F)** shows the flap after inset and de-epithelialization.

**Figure 3:**
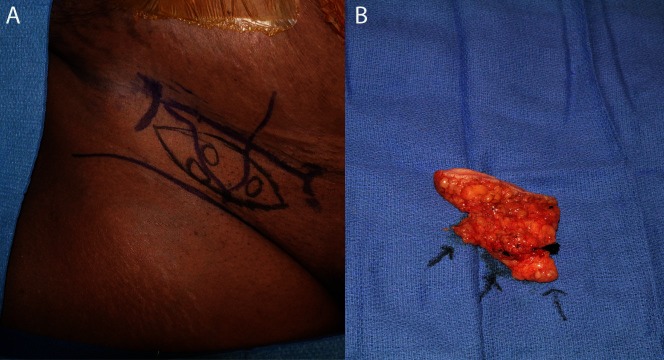
Skin markings for the groin-based lymphatic flap with nodes identified by lymphoscintigraphy and ICG **(A)**. The dissected flap is shown with arrows indicating lymph nodes **(B)**.

Beyond LVA, VLNT has also been successfully performed for the treatment of lymphedema. In VLNT, a free tissue flap containing vascularized lymph nodes is transferred from one of various donor sites around the body to either a soft tissue defect or a non-anatomic area on the edematous limb in order to reconstitute lymphatic flow (1, 40, 42). Commonly used donor sites and associated lymph nodes include the supraclavicular (Figure 2), groin (Figure 3), submental, axillary, and thoracic flaps (42). The use of supermicrosurgery allows for the transfer of lymphoadipose flaps with small perforating vessels less than 0.8 mm in caliber, as well as manipulation of venules and lymphatic vessels within or near the implanted tissue (4, 43). Supermicrosurgery has been increasingly used for VLNT, as the technique permits the use of thinner flaps to reduce donor-site morbidity and may improve lymphatic drainage (44).

Two theories currently exist for how VLNT treats lymphedema. One theory is that lymphangiogenesis occurs via growth factors produced by the transplanted lymph nodes, and that newly sprouted lymphatic vessels are able to reconstitute lymphatic pathways (1, 45). Another theory posits that the transferred vascularized lymph node acts a lymphatic pump: the combination of pulsations at the arterial anastomosis acting as a pump and the suction effect of the flap’s venous output create a driving force for lymphatic drainage through the transplanted lymph node (1, 44, 46). The lack of current histological, imaging or physiologic proof of these mechanisms in action prevents definitive validation of either theory. However, Chong et al. have recently developed a method for visualizing lymphatic vessel contractility in mice through the use of infra-red imaging, which may be used to uncover the underlying mechanism behind VLNT in the future (47).

Many studies have validated the use of simple microsurgical VLNT for the treatment of lymphedema, with some showing superior outcomes over LVA (1, 48–51). Despite its efficacy, VLNT does not always result in optimal lymphatic outflow and also constitutes free flap surgery, which can involve significant donor-site morbidity. However, advancement and refinement in technique aim to reduce these drawbacks. Visconti et al. have described the successful use of a new cervical lymph node-adipo-cutaenous flap in rats using supermicrosurgical perforator-to-perforator anastomosis, and Yamamoto et al. have recently reported the use of VLNT using both perforator-to-perforator anastomosis and efferent lymphaticolymphatic anastomosis between donor and recipient lymphatic vessels (52, 53). Moreover, some surgeons combine LVA with VLNT, anastomosing efferent lymphatic vessels to outflow veins within or near the flap in order to improve lymphatic drainage of the implanted tissue (52). Continued experimentation with techniques to improve lymphatic drainage and additional research on optimal donor and recipient sites will likely improve patient outcomes in the future and support more routine use of this procedure for the treatment of lymphedema.

### Soft Tissue Reconstruction

Although functional and aesthetic outcomes have improved with the use of perforator flaps for microsurgical free tissue reconstruction, the procedure still risks compromising the major vessels, involves long operating times, and can cause significant donor-site morbidity and scarring (54–56). The supermicrosurgical technique of raising a true perforator flap and performing perforator-to-perforator anastomosis at the recipient site can help solve many of these issues. As a result, supermicrosurgery has been increasingly used in lower extremity, craniofacial, hand, and breast reconstruction (17, 19, 57, 58).

**Figure 4:**
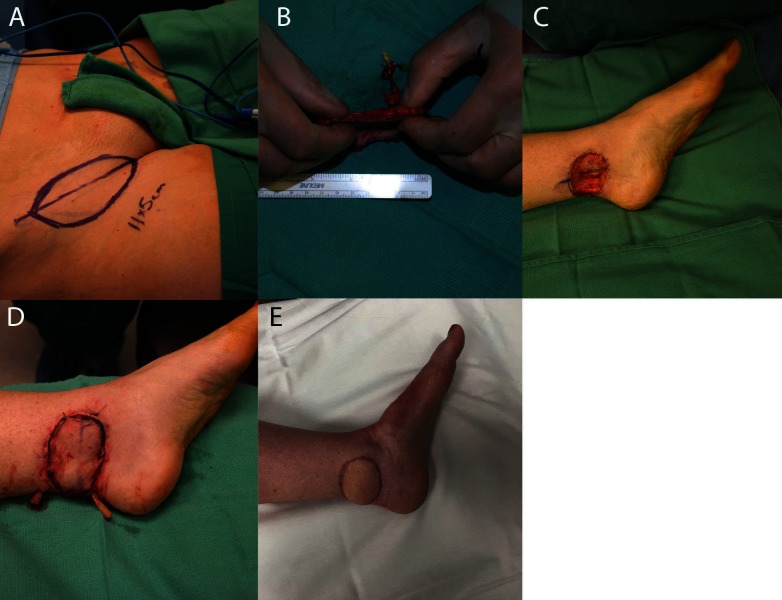
**(A)** shows markings for the SCIP flap, and **(B)** shows the thin dissected flap. **(C)** demonstrates the recipient wound bed, and **(D)** demonstrates the recipient site with the flap in place. **(E)** shows the same foot 3 months postoperatively.

The main advantage of supermicrosurgical reconstruction is that it allows for the use of *true* perforators for anastomosis, defined by Wei et al. as cutaneous vessels that pierce the deep fascia on their course to ultimately reach the skin (59). The utilization of a supermicrosurgical approach creates a very thin flap by dissecting only the most superficial perforators; Figure 4 demonstrates the use of a thin Superficial Circumflex Iliac Perforator (SCIP) flap to reconstruct a small defect over the Achilles tendon. These thin true perforator flaps reduce donor-site morbidity, as cutting in a suprafascial plane helps preserve muscle, fascia, cutaneous nerves and major vessels (60). Additionally, since the pedicles for these true perforator flaps are shorter, dissection is less time-consuming and time spent in the operating room is reduced (4). Finally, the elucidation of the perforasome theory, mapping of perforators throughout the body, and the utilization of preoperative imaging for locating perforators have provided surgeons with much greater flexibility in regards to choosing a specific donor site that best suits the reconstruction (54). Several kinds of perforator flaps harvested for supermicrosurgical anastomosis have been described, including the thoracodorsal, lateral thoracic, superficial circumflex iliac, paraumbilical, anterolateral thigh and gluteal artery perforator flaps (60).

**Figure 5:**
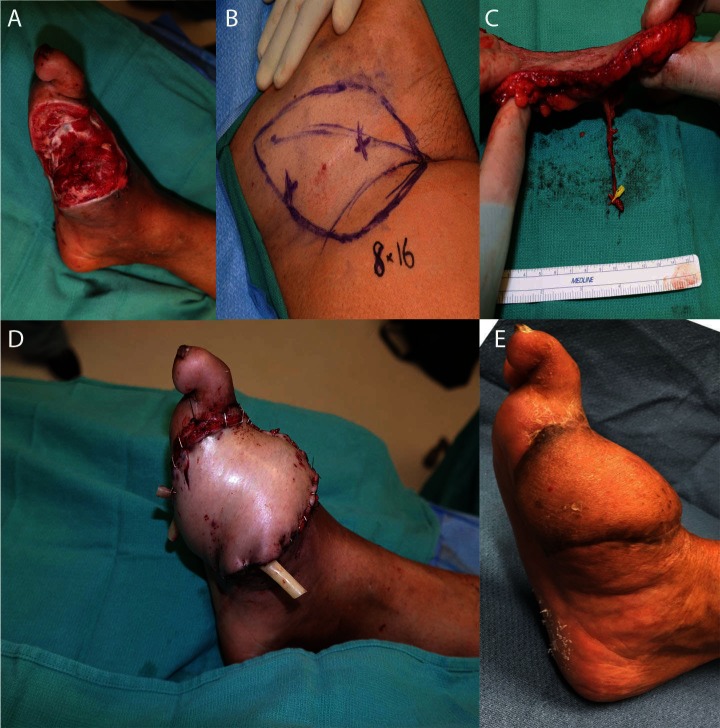
Salvage of a diabetic wound. The patient presented with an infected great toe amputation, and underwent 3 staged debridements with osteotomy. **(A)** shows the defect before soft tissue reconstruction, **(B)** demonstrates the markings for SCIP flap, **(C)** shows the dissected flap and vessels, and **(D)** shows the flap after inset. **(E)** demonstrates the flap 6 months postoperatively.

The use of a perforator as a recipient vessel also offers several advantages. First, the large number of perforators available within even small regions of the body allows for an increase in selection of choices for a recipient vessel (15). This is particularly useful when the larger vessels are present within the zone of injury, or when thorough dissection would be required to expose a deep vessel, thus increasing the risk of recipient-site morbidity. Perforator-to-perforator anastomosis is also useful for patients with significant atherosclerosis or other conditions resulting in fewer major vessels supplying the leg. For example, supermicrosurgery has been increasingly utilized in diabetic foot reconstruction by using the collateral vessels in the zone of ischemia as recipient vessels (7, 61). In this case, recipient perforators may be used to perform free flap coverage without compromising distal flow (Figure 5). Finally, perforator-to-perforator anastomosis also has implications for flap salvage, providing additional options for bypassing areas of congestion or thrombosis (23, 62, 63).

Despite the benefits offered by supermicrosurgical soft tissue reconstruction, the technique has its limitations. First, there is a presumed increased probability of developing thrombosis of the small perforator vessels, which predisposes to flap necrosis (64). Additionally, many institutions are not equipped with the technology required to perform supermicrosurgery, including powerful microscopes capable of 50x magnification, fine surgical instruments, and imaging technologies necessary for perforator mapping, including multidetector-row computer tomography, magnetic resonance imaging, and near-infrared fluorescence imaging with indocyanine green (65–67). Finally, the technique takes significant time to master due to the short length of the flap pedicle and the difficulty in preforming anastomosis of small vessels.

### Hand Surgery

One area that has received substantial attention in supermicrosurgical research is hand surgery. Supermicrosurgery is ideal for reconstructions of the hand, as the delicate anatomic structure of fingertips necessitates the use of very fine surgical instruments and sutures. Furthermore, for safe reconstruction of the distal digits, supermicrosurgical skills are required, as the vessel calibers in this location are typically smaller than 0.5 mm (68). Supermicrosurgery has been successfully used for fingertip replantation, toe-tip transfers, free nail flaps and free perforator flaps (24, 68–70).

With the advent of supermicrosurgery, fingertip replantation is now used in digital amputations in order to permit the restoration of function, length and aesthetics while avoiding painful stump neuromas (71). Positive outcomes for fingertip replantation have been validated in the literature; for example, a study by Hattori et al. found that patients with successful replantation had a greater range of motion, less pain, better disabilities of the arm, shoulder and hand (DASH) score, and were more satisfied compared to the patients undergoing amputation and revision (72). Still, many hand surgeons perceive that these technically challenging, lengthy procedures provide little functional improvement over revision amputations, and the success rate of replantations may decrease in crush injuries in which the amputated fingertips are severely damaged (68).

Fingertip reconstruction with free tissue flaps, including various perforator, nail and toe flaps has also been made possible through the use of supermicrosurgery (69, 73, 74). These procedures are usually reserved for situations where the distal fingertip is not available or too severely injured to be used for replantation. The development and refinement of new flaps, such as the dorsoradial perforator (DRAP), superficial palmar branch of the radial artery (SUPBRA), and medialis pedis free flaps, have allowed for a single stage fingertip reconstruction under regional anesthesia with minimal donor site scarring. These perforator flaps are innervated and can restore some sensory function, as well as preserving aesthetic outcomes including skin texture and color (69, 75, 76). As supermicrosurgery becomes increasingly incorporated into the field of hand surgery, the introduction of additional flaps and improvement of current techniques may allow for flap reconstruction to completely restore digital function in cases where replantation is not possible.

### Other Applications

A major benefit of supermicrosurgery is the ability to manipulate structures on a much finer scale, allowing for precise and organic reconstructions of very delicate body parts. For example, successful replantation of partially amputated nasal segments have been performed with high rates of success (77, 78). Additionally, reconstruction of the upper eyelid has been performed using an ear helix flap through supermicrosurgical techniques, and successful tongue reconstruction has also been previously reported using supermicrosurgery (18, 79). Finally, urethral reconstructions may be performed with supermicrosurgery, using either appendix transfers or more traditional free flaps (80, 81). These procedures highlight the importance of supermicrosurgical procedures in further expanding the reaches of microvascular surgery, allowing for virtually every part of the body to be successfully reconstructed.

Microvascular aesthetic surgery has also been enriched with the advent of supermicrosurgical techniques. In particular, perforator adiposal free flaps have been well-described for the use of facial and breast augmentation, with aesthetic outcomes reportedly greater than free fat injection and the conventional dermal fat flap (5). The use of flaps for aesthetic procedures also affords the surgeon significant flexibility in the procedure; for example, the paraumbilical perforator adiposal flap for breast reconstruction can be easily defatted if the bulky fat needs to be reduced, a variety of perforating vessels may be used for this technique, and many flaps can be raised and tailored to the specific needs of patients depending on the shape and size of the defect (82). According to Koshima et al., utilizing supermicrosurgery to raise a paraumbilical perforator flap for facial and breast augmentation is particularly useful in young women who may become pregnant in the future, due to minimal donor-site morbidity of the abdomen, and for children who have the potential for further skeletal growth (82).

Supermicrosurgery has also contributed to innovations in microneural repair. In 2010, Koshima et al. introduced a new method for peripheral nerve repair utilizing supermicrosurgical techniques, which he called the fascicular turnover flap. The procedure first involves the separation and coaptation of a single nerve fascicle from either the proximal or distal end of a nerve gap. The nerve fascicle is then turned over the nerve gap and positioned against the contralateral nerve end, and the fascicular flap and contralateral nerve trunk are sutured. For larger nerve gaps, bilateral turnover fascicular flaps from both the proximal and distal nerve trunks can be sutured along the middle portion of the nerve gap (83). It is believed that axonal flow is eventually reconstituted via axonal sprouting (83, 84). Although no controls were used in their experiments, Koshima et al. reported excellent sensory recovery for the fascicular turnover flap in digital nerve repair, as well as a quicker operating time and less invasive procedure compared with the vascularized nerve graft method (4, 5, 83, 85).

## Training in Supermicrosurgery

Although most plastic surgery residency programs teach microsurgical techniques, supermicrosurgery requires a higher skill level for eye–microscope–hand coordination, more dexterous handling of tissues, and more refined motor skills than microsurgery (86). These technical skills need to be trained and extensively practiced in order to be appropriately utilized in the operating room.

Surgical simulation training aids in the development of critical psychomotor, technical and judgment skills through repetition, and by providing the opportunity to learn from one’s errors without causing major harm (87). As supermicrosurgery is a new technique practiced by a minority of surgeons, there is an increased need for surgical simulators and training models to facilitate rapid acquisition of skills. Currently, several simulators exist for training supermicrosurgical techniques, including synthetic and biologic nonliving simulators, as well as live animal models (64).

Synthetic models are recommended for acquiring fundamental supermicrosurgical skills, such as learning microscopic adjustments and the handling of surgical tools. The most basic models include practice cards containing affixed silicon tubes measuring less than 0.8 mm in diameter which may be used for practicing anastomosis of supermicrosurgery-caliber vessels (88). These practice cards are attached to the stage of a microscope, and can be manipulated with the microsurgical instruments that are used clinically. More high-fidelity biologic cadaveric models, such as the chicken thigh model introduced by Chen et al., can be used for advanced skill acquisition once basic skills have been achieved and the trainee is comfortable using the microscope and associated instruments (86). In the chicken thigh model, branches of the ischiatic artery and vein have diameters ranging between 0.3–0.5 mm, allowing for practice on biologic submillimeter vessels. A major benefit of nonliving biologic models is that the trainee can receive tactile feedback that is nearly identical to procedures performed in human patients. These models are cost-effective and do not require pre- or postoperative care (86).

Once supermicrosurgical skills have been sufficiently developed using nonliving models, the trainee can progress to practicing on live animal models. Several living rat models featuring an assortment of submillimeter vessels are available for the practice of supermicrosurgical techniques (89–92). Different models allow for the practice of a large variety of techniques, ranging from simple perforator-to-perforator anastomoses to complete flap transfers. Once the surgeon is proficient at performing these skills on live animal models, he or she may be ready to utilize these techniques in the care of patients.

## Robotic-Assisted Surgery as an Enabling Technology

One of the greatest challenges with supermicrosurgery is the high level of dexterity and surgical control required for successful anastomosis. Robotic-assisted surgery (RAS), defined as surgery that is performed by a human surgeon through the use of a robotic instrument, may facilitate use of this technique. Although RAS is already used to perform very delicate surgeries requiring manipulation of small structures, there is currently only 1 study in the literature describing a successful supermicrosurgical procedure performed with the Da Vinci surgical system (93).

RAS offers several unique benefits that may assist surgeons with performing the technique and enhance supermicrosurgical procedures. First, the Da Vinci allows highly precise control of instruments that may be continuously adjusted throughout the procedure, which increases the exactness of the operator’s movements. Additionally, the Da Vinci surgical system allows the surgeon to work on anatomic areas that may be difficult to access in conventional surgery; this may offer the surgeon greater ergonomic comfort during surgery and allows for additional operating angles. Finally, RAS allows for complete repression of ordinary hand tremors, further increasing the precision of surgical movements (93). Therefore, RAS enables accessibility to the utilization of supermicrosurgery.

However, use of RAS requires increased operating time, is expensive, and the repertoire of movements and actions performed through RAS are more limited than those that can be performed by human hands. Therefore, the use of RAS by experienced surgeons will likely be reserved for procedures that require extremely delicate supermicrosurgical skills beyond those needed for more routine procedures, and for performing remote-controlled surgeries from distant sites (93). Nonetheless, RAS represents an exciting new frontier for supermicrosurgery and may be the key to manipulating even smaller anatomic structures in the future.

## Future Directions

### Organ Transplantation

Studies are underway investigating limb transplantation, uterine transplantation, and eye transplantation in animal models using supermicrosurgical techniques (92, 94, 95). Kisu et al. have recently reported the use of supermicrosurgery in a successful uterine transplantation in cynomolgus monkeys through anastomosis of the internal iliac artery and superficial uterine vein with their corresponding recipient vessels (94). The success of this procedure highlights the importance of supermicrosurgery for transplantation of organs with small feeding vessels that were previously not amenable to microsurgery. A similar principle was illustrated in the limb transplantation procedures performed in a rat animal model by Sucher et al. (92). It is perceivable that bioengineered body parts could also rely heavily on supermicrosurgical techniques in the future.

### Customized Reconstruction

An exciting consequence of the increased use of supermicrosurgery in soft tissue reconstruction will be the possibility of raising customized flaps tailored to fit individual reconstructive needs. Since supermicrosugery allows surgeons to use thin flaps from all over the body with minimal donor-site morbidity, it is becoming increasingly possible to design flaps based on the specific color, size, three-dimensional shape, blood supply and innervation patterns desired. Therefore, further refinement of supermicrosurgical procedures and experimentation with new flap types may allow surgeons to perform highly customized reconstructions that improve both aesthetic and functional outcomes in patients with complex reconstructive needs (4).

The concept of “like-with-like” reconstruction has already begun to gain traction with the advent of perforator flaps, but supermicrosurgery opens the doors for truly individualized free-style perforator flaps based on needs of the reconstruction. There are a few studies in the literature describing experimentation with customized perforator flaps designed to exactly reconstruct soft tissue defects (96, 97). For example, Jin et al. have recently reported their experience using supermicrosurgery to perform like-with-like buccal reconstruction with the SCIP flap; the authors report designing flaps with surface areas and volumes tailored to fulfill both the cosmetic and functional requirements of the recipient oral defect. Using this procedure, the authors described excellent aesthetic and functional outcomes in the buccal region, successful like-with-like reconstruction, and minimal donor-site scarring (96).

Stacked perforator free flaps represent another opportunity for supermicrosurgery to solve complex reconstructive problems. “Stacking” of flaps involves the transfer of tissue from multiple donor sites into a single recipient site, a technique which has previously been described for breast reconstruction in patients that require additional volume to achieve an optimum result. Stacked deep inferior epigastric perforator (DIEP) flaps, profunda artery perforator flaps, superficial inferior epigastric artery (SIEA) flaps, and several other combinations have been used for this purpose (98, 99). Although these procedures have generally produced successful reconstructive results, they still involve significant recipient and donor-site morbidity, especially if bilateral donor sites are used. Supermicrosurgery may enhance the utility of stacked flaps by allowing more freedom in choosing donor and recipient vessels for anastomosis; for example, one can envision using small intercostal or internal mammary perforators as recipient vessels for stacked perforator flap inset using perforator-to-perforator anastomosis, or using the feeding perforating vessel of the primary free flap as the recipient vessel for a second flap through intra-flap anastomosis (99–102). These techniques may reduce donor and recipient site morbidity by eliminating the need for large vessel dissection.

Supermicrosurgery may also be applied to the concept of chimeric flaps, which consist of separate flaps of various different tissue types that are connected to one another through a single vascular pedicle. They can be further broken down into those chimeric flaps which are connected by their intrinsic vasculature and prefabricated flaps which are connected through microanastomosis. By allowing for the transfer of different tissue types and the independent inset of each component, chimeric flaps create a large degree of spatial freedom in order to restore volume and contour of complex 3-dimensional defects (103). Supermicrosurgery allows for the dissection and use of unique intrinsic chimeric flaps relying on small feeding vessels that are not amenable to traditional microsurgery in order to reconstruct complex defects; for example, Yamamoto et al. have previously described the use of a quadruple-component intrinsic chimeric SCIP flap that included portions of the Sartorius muscle, deep fascia, inguinal lymph node and skin/fat for a complex ankle reconstruction (104). Fernandez Garrido et al. similarly described the use of a chimeric SCIP flap with a piece of external oblique muscle fascia tailored to reconstruct a dorsal foot defect (105). With further refinement of technique, supermicrosurgery may allow for the prefabrication of highly customized, multi-component chimeric flaps using tissues from all over the body, without the limitation of feeding vessel size, that can be precisely tailored to a patient’s reconstructive needs.

### Extreme Salvage

An important application of supermicrosurgery has been the ability to salvage extremities in the setting of significant systemic or local disease. With the use of supermicrosurgical techniques, extreme limb salvage can be performed in the setting of conditions that have previously limited simple microsurgical free tissue transfer, including renal failure, post-transplant patients on immunosuppressant medications, severe diabetic microvascular disease, advanced peripheral vascular disease with severe ischemia, and a history of amputation. In these cases, wound healing is impaired, circulation is poor and major vessels are often calcified, making microsurgery extremely difficult (61, 106). Through supermicrosurgery, small collateral vessels that have formed in the setting of chronic ischemia, or small perforators left over after major lower extremity trauma, can be used as recipient vessels for free tissue anastomosis without compromising distal flow (19, 61). Thus, even extremely sick patients with poor vascular flow from the major vessels due to injury or advanced ischemia can be given the opportunity for reconstruction as long as they have a small artery with an adequate pulse. For example, Suh et al. showed that patients with ischemic diabetic foot ulcers and significant comorbidities, including renal failure, immunosuppression, and previous amputation, can nonetheless undergo lower extremity reconstruction using small recipient end arteries or perforators in a different angiosome territory, with a reported overall limb salvage rate of 90.5% (7). Supermicrosurgery presents the best option for patients with severe injuries and multiple comorbidities to avoid extremity amputation.

## Conclusions

Supermicrosurgery has greatly expanded the scope of procedures that can be performed under a microscope. Since the anastomosis of small vessels is a central component of many surgeries, supermicrosurgery has had an immense impact on a wide variety of different procedures, from lymphedema management to soft tissue reconstruction, digit replantation and aesthetic surgery. Importantly, supermicrosurgery is a brand new field, and a consensus was only met on its name in 2010 (4). Therefore, it is certain that the field will continue to see additional innovations, refinements and adoption in the coming years. The goal is that with further innovations and refinement of technique, the supermicrosurgical armamentarium will expand, operating times will shorten, the learning curve will decrease and patient outcomes will improve.

There is now a need for outcome-based research investigating functional outcomes and patient satisfaction following supermicrosurgery. Using the results of such studies, it will be possible to formally adopt supermicrosurgery in clinical decision-making algorithms when facing challenging cases in reconstruction. The development of additional simulation models and training programs dedicated to supermicrosurgery will be necessary to increase widespread adoption of these techniques.

## Author Contributions

IB, DG, and KP all contributed to the concept, literature review, generation of figures, manuscript drafting and editing of this review. All authors verified the contents of the final manuscript and maintain that the information contained therein is accurate and true.

## Conflict of Interest Statement

The authors declare that the research was conducted in the absence of any commercial or financial relationships that could be construed as a potential conflict of interest.
